# Comparative Study on Carbon Erosion of Nickel Alloys in the Presence of Organic Compounds under Various Reaction Conditions

**DOI:** 10.3390/ma15249033

**Published:** 2022-12-17

**Authors:** Alexander M. Volodin, Roman M. Kenzhin, Yury I. Bauman, Sofya D. Afonnikova, Arina R. Potylitsyna, Yury V. Shubin, Ilya V. Mishakov, Aleksey A. Vedyagin

**Affiliations:** 1 Boreskov Institute of Catalysis SB RAS, 5 Lavrentyev ave., 630090 Novosibirsk, Russia; 2 Department of Natural Sciences, Novosibirsk State University, 2 Pirogova str., 630090 Novosibirsk, Russia; 3 Nikolaev Institute of Inorganic Chemistry SB RAS, 3 Lavrentyev ave., 630090 Novosibirsk, Russia

**Keywords:** nickel alloys, carbon erosion, carbon nanofibers, ferromagnetic resonance, RAPET

## Abstract

The processes of carbon erosion of nickel alloys during the catalytic pyrolysis of organic compounds with the formation of carbon nanofibers in a flow-through reactor as well as under reaction conditions in a close volume (Reactions under Autogenic Pressure at Elevated Temperature, RAPET) were studied. The efficiency of the ferromagnetic resonance method to monitor the appearance of catalytically active nickel particles in these processes has been shown. As found, the interaction of bulk Ni-Cr alloy with the reaction medium containing halogenated hydrocarbons (1,2-dichloroethane, 1-iodobutane, 1-bromobutane) results in the appearance of ferromagnetic particles of similar dimensions (~200–300 nm). In the cases of hexachlorobenzene and hexafluorobenzene, the presence of a hydrogen source (hexamethylbenzene) in the reaction mixture was shown to be highly required. The microdispersed samples of Ni-Cu and Ni-Mo alloys were prepared by mechanochemical alloying of powders and by reductive thermolysis of salts-precursors, accordingly. Their interaction with polymers (polyethylene and polyvinyl chloride) under RAPET conditions and with ethylene and 1,2-dichloroethane in a flow-through reactor are comparatively studied as well. According to microscopic data, the morphology of the formed carbon nanofibers is affected by the alloy composition and by the nature of the used organic substrate.

## 1. Introduction

Carbon nanomaterials of various functional destinations are widely applied in modern science and technology [1,2]. Among the variety of these materials, two classes, carbon nanotubes (CNT) and carbon nanofibers (CNF) should be mentioned especially. Both CNT and CNF can be derived from hydrocarbon sources via catalytic methods [3,4,5,6,7]. As a rule, the catalytic growth of carbon nanostructures takes place over the dispersed particles of iron subgroup metals [8,9,10]. In particular, nickel and its alloys are of special interest [11,12,13,14,15,16,17,18,19,20,21].

It should be noted that the dispersed metal particles are easily oxidized being in contact with the air. Therefore, in order to obtain such dispersed particles, oxide precursors are often deposited on the supports with a high surface area (Al_2_O_3_, MgO, SiO_2_, zeolites, etc.) and then reduced in a hydrogen flow immediately before the catalytic procedures [13,14,15,22,23]. One of the important issues is that only the dispersed particles of metals and alloys possess catalytic activity in the process of catalytic carbon growth. This process is also known as catalytic chemical vapor deposition (CCVD). The size and the chemical composition of the dispersed particles affect significantly the efficiency of the carbon nanostructures formation as well as their morphology. The commercially available bulk metals and alloys (foils, wires, and rods) produced on an industrial scale do not exhibit such a catalytic activity. One of the possible ways to form catalytically active particles from the bulk metal items is an initiation of the catalytic corrosion processes over their surface due to interaction with the reaction (aggressive) medium. Finally, the corrosion processes result in nano-/micro-structuring of the surface (the formation of the surface dispersed particles) or even complete disintegration of the initial items. The first scenario is well-known for the reductive-oxidative catalytic reactions [24,25,26], which are accompanied by the significant loosening of the metal surface and the formation of dispersed particles of a wide size distribution on it.

On the other hand, all the metals (Fe, Co, and Ni) traditionally used for the synthesis of nanostructured carbon possess ferromagnetism [27,28,29,30]. Therefore, the ferromagnetic resonance (FMR) method is applicable, in principle, to study the dispersed particles of these metals within the composition of catalysts. Despite many attempts by researchers to use this technique for catalyst characterization, it did not get a wide application in practice. Moreover, it was quite rarely applied to explore the catalysts for CNF synthesis. All this is connected with the difficulties in the interpretation of FMR spectra, which are caused by the presence of ferromagnets of high-spin states along with strong exchange interactions characterized by a high anisotropy. The most important factor complicating the application of FMR is the absence of any information regarding the spin of the studied samples that do not allow making even a qualitative estimation of the concentration of the ferromagnetic particles in the sample, contrary to the paramagnetic sites registered in different catalytic systems by the electron paramagnetic resonance (EPR) technique.

Nevertheless, as we have reported recently [31,32,33,34], taking into account the features of chemical and magnetic properties of the nickel-containing catalysts, the FMR method can be successfully applied to monitor the appearance of the dispersed metal particles during the catalytic reaction and to observe the noticeable changes in the stoichiometry of the alloys being used. The main feature of the catalytic systems described in these works deals with the phenomenon of self-organization of the catalyst when the relatively uniform in size metal particles (~200 nm) are being formed from different bulk metal precursors. Further, these particles act as sites for the catalytic growth of CNF. The appearance of such submicron ferromagnetic particles characterized by a narrow size distribution allows applying the FMR technique not only to detect them but to estimate the kinetics of their accumulation during the catalytic processes as well.

The FMR method is also useful to study the self-disintegration processes of nickel alloys in a closed volume in an in situ mode. Such a mode is known as Reactions under Autogenic Pressure at Elevated Temperature (RAPET). It was originally proposed by Prof. A. Gedanken et al. in their numerous works [35,36,37,38,39,40]. The RAPET approach allows studying the dynamics of the processes and revealing the experimental conditions required for the synthesis of a series of a new type of structured inorganic nanomaterials, which appeared as intermediates during the processes of catalytic transformation of various classes of organic molecules [31,32,33].

The present work is aimed to further develop this scientific direction and to widen the number of catalytic systems studied by means of FMR and used to synthesize CNF. The interaction of a wide range of organic substrates with nickel alloys was examined in a flow-through reactor and under RAPET conditions. In both cases, the FMR method was applied to monitor the appearance of dispersed nickel-containing particles. The resulting carbon nanostructures were investigated by electron microscopic techniques.

## 2. Materials and Methods

### 2.1. Chemicals and Materials

Nichrome wire (~75.5% Ni, ~23% Cr, ~1.5% Fe) of 0.1 mm in diameter was purchased from VZPS (Vladimir, Russia) and used without any further purification. Nickel powder (RusRedMet, Saint-Petersburg, Russia) and copper powder (NMK-Ural, Yekaterinburg, Russia) were used to synthesizing the Ni-Cu alloy.

NiCl_2_·6H_2_O (Reachem, Dzerzhinsk, Russia) was used to synthesize [Ni(NH_3_)_6_]Cl_2_. A total of 5 g of NiCl_2_·6H_2_O was dissolved in a minimal amount of distilled water. About 30 mL of concentrated NH_3_ along with 10 mL of saturated NH_4_Cl solution was added to the solution of nickel chloride. The slow formation of a lilac precipitate was observed. The solution was maintained for 30 min. Then, the precipitate was filtered using a glass filter and washed with ethanol.

(NH_4_)_6_Mo_7_O_24_∙4H_2_O was purchased from Reachem (Dzerzhinsk, Russia). All the chemicals were of chemical purity grade and were used without any preliminary purification.

### 2.2. Synthesis of Alloy Samples

The Ni-Cu alloy containing 12 wt.% of copper was prepared by the mechanochemical alloying (MCA) method as described elsewhere [41]. The preliminarily prepared mixture of nickel and copper powders (10 g) with a weight ratio Ni/Cu of 88/12 was loaded into stainless steel jars of 250 mL in volume along with stainless steel grinding balls (340 g). The diameter of the grinding balls was 5 mm. The MCA procedure was performed using a planetary mill Activator 2S (Activator LLC, Novosibirsk, Russia). The rotation frequency rate of the platform (956 rpm) and the jars (449 rpm) was controlled using an industrial frequency inverter VF-S15 (Toshiba Schneider Inverter Corp., Nagoya, Japan). The estimated acceleration of the grinding balls was 784 m/c^2^ (~80 G). The jars were cooled with water in order to avoid overheating during the MCA procedure. Finally, the Ni-Cu alloy sample was unloaded in the air, separated from the grinding balls using a sieve, and weighed. The sample was labeled as Ni-Cu(12%).

The Ni-Mo alloy was prepared by the high-temperature reductive thermolysis of a multicomponent precursor. The latter was obtained as follows. The joint concentrated solution of [Ni(NH_3_)_6_]Cl_2_ and (NH_4_)_6_Mo_7_O_24_, containing the calculated amounts of nickel and molybdenum to obtain 1 g of alloy, was added at intense stirring to a large volume of acetone cooled down to 0 °C. Due to the low solubility of these salts in acetone, the formation of an oversaturated solution leading to immediate precipitation of the microheterogeneous mixture of precursors occurs. The precipitate was filtered, dried in the air for ~20 h, and calcined in a hydrogen flow (130 mL/min) at 800 °C for 1 h. The temperature ramping rate was 20 °C/min. Then, the sample was cooled down in a hydrogen atmosphere and purged with helium. The Ni/Mo weight ratio was 92/8. The sample was labeled as Ni-Mo(8%).

### 2.3. Characterization of Alloys and Carbon Samples

Both the alloys and the carbon samples were studied by scanning electron microscopy (SEM). The images were collected using a JSM-6460 electron microscope (JEOL, Tokyo, Japan) at a magnification from 1000× to 100,000×.

The morphology of the carbon product was explored by transmission electron microscopy (TEM) using a Hitachi HT7700 TEM (Hitachi High-Technologies Corp., Tokyo, Japan) working at an acceleration voltage of 100 kV and equipped with a W source.

The low-temperature nitrogen adsorption method (Brunauer–Emmett–Teller, BET) was used to determine the specific surface area (SSA) of the samples. The samples were preliminary degassed under an oil-free vacuum at 300 °C for 5 h. The adsorption/desorption isotherms were recorded at 77 K on an ASAP-2400 automated instrument (Micromeritics, Norcross, GA, USA).

The ferromagnetic resonance (FMR) spectra were registered at room temperature using an ERS-221 spectrometer (Center of Scientific Instruments Engineering, Leipzig, Germany). The initial alloys as well as the samples after catalytic experiments in a flow-through reactor were studied. A specimen (0.3–0.5 mg for alloys, 5–10 mg for carbon samples) was loaded into a quartz ampule. Such a low sample loading is stipulated by an intensive absorption of microwave power by metals. The carbon samples were homogenized in an agate mortar before loading into the ampule.

### 2.4. Carbon Erosion of Alloys in a Flow-Through Reactor

The process of carbon erosion of the alloys followed by the growth of carbon nanofibers was studied in a flow-through reactor equipped with McBain balance, which allowed registering the carbon accumulation in real-time mode. A specimen of the alloy (~2 mg) was loaded into a quartz basket, which was then placed inside the reactor. Then, the reactor was purged with argon to eliminate any oxygen traces and heated up to the desired temperature in a range from 450 to 750 °C. The heated reactor was fed with hydrogen to reduce the catalyst (until the constant weight of the sample). After the reduction stage, the reactor was fed with the reaction mixture containing 1,2-dichloroethane or ethylene (6 vol%), hydrogen (56 vol%), and argon (38 vol%). The total flow rate of the reaction mixture was 267 mL/min. The duration of the experiments was 2 h. The weight of the sample was recorded every 2 min. Finally, the reactor was cooled down in an argon flow. The sample was unloaded, weighed, and characterized.

### 2.5. Carbon Erosion of Alloys under RAPET Conditions

**Figure 1:**
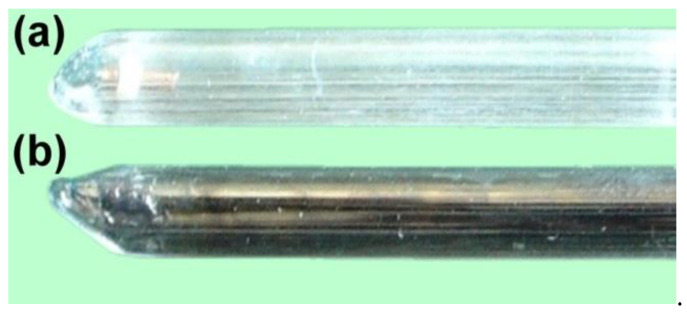
Quartz ampules used for the FMR studies: (**a**) initial ampule loaded with the sample (a piece of nichrome wire) and organic substrate (1,2-dichloroethane); (**b**) ampule after the RAPET experiment.

In RAPET experiments, the following organic substrates were used: 1,2-dichloroethane (DCE); 1-iodobutane; 1-bromobutane; hexachlorobenzene (HCB); hexafluorobenzene (HFB); hexamethylbenzene (HMB); polyethylene, and polyvinyl chloride. The alloy sample (0.2–0.3 mg) along with organic substrate (2–3 mg) were loaded into the quartz ampule presented in Figure 1. The ampule was sealed. The FMR spectra were recorded before and after the ampule was heated to the desired temperature. After the experiment and the registration of the FMR spectrum, the ampule was opened, and the sample was characterized by microscopic techniques.

As already mentioned, the registration of the FMR spectra was carried out at room temperature. Under such conditions, only materials with a Curie temperature above room temperature can possess ferromagnetism. Table 1 shows the reference data on the Curie temperature for pure nickel and for the nickel alloys used in the present study.

## 3. Results and Discussion

### 3.1. Interaction of Ni-Cr Alloy with Halogenated Hydrocarbons in a Flow-Through Reactor

**Figure 2:**
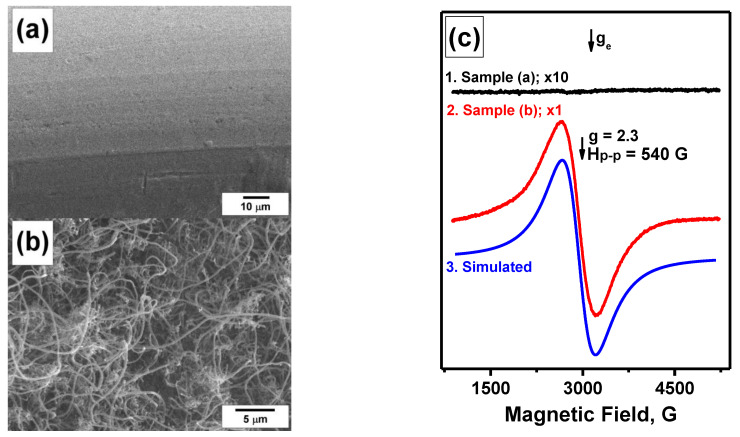
SEM images of the initial nichrome wire (**a**) and the carbon product obtained via the catalytic pyrolysis of DCE over this nichrome wire (**b**) and corresponding FMR spectra (**c**). Note that the spectra are normalized in intensity with regard to the nickel content in the samples. The spectrum of sample (**b**) is simulated with Lorentzian line shape and *H*_p-p_ = 540 G. The multipliers ×1 and ×10 indicate the relative amplification coefficients used at registering the spectra.

It is evident that nickel alloys, not possessing ferromagnetism at the temperature of the spectra registration (in our case, room temperature), should not give any FMR signals. In this regard, the FMR method was recently shown to give a possibility for monitoring the evolution of the initial alloys during the catalytic synthesis of CNF [31]. Here, the bulk Ni-Cr alloy (a piece of wire) was examined before and after its interaction with the reaction medium containing DCE vapors. Figure 2a,b present typical SEM images of the initial alloy sample and the carbon product accumulated in a flow-through reactor at 650 °C for 2 h, accordingly. The corresponding FMR spectra are shown in Figure 2c. The submicron metal particles contained in the carbon product are characterized by an intensive FMR signal at room temperature. This testifies to the appearance of ferromagnetism in them due to a decrease in chromium concentration and a rise of the Curie temperature above room temperature (Table 1). The observed FMR spectrum can be appropriately fitted by a single Lorentzian-shaped line (see the simulated spectrum in Figure 2c). This indicates the narrow size distribution of the ferromagnetic particles in the studied sample and agrees well with the recently reported microscopic data regarding the character size of 200–300 nm for the similarly obtained particles [31].

Similar situation was reported for the other bulk nickel alloys (chromel, alumel, etc.). Despite the difference in the shape of the FMR spectra of the initial alloys, they are uninformative ones, and, therefore, their interpretation is complicated. At the same time, after their contact with the aggressive reaction mixture and the formation of carbon nanostructures, the FMR spectra undergo noticeable changes. The wide singlet lines with *g* = 2.3, which is typical of the dispersed nickel particles, appeared [9,31]. It should be noted that in these works, the shapes of the FMR spectra cannot be fitted by a single Lorentzian-shaped line. This is presumably connected with a wider size distribution of the ferromagnetic particles in the cases of the described samples if compared with the sample obtained via the self-disintegration of nichrome (Figure 2).

**Figure 3:**
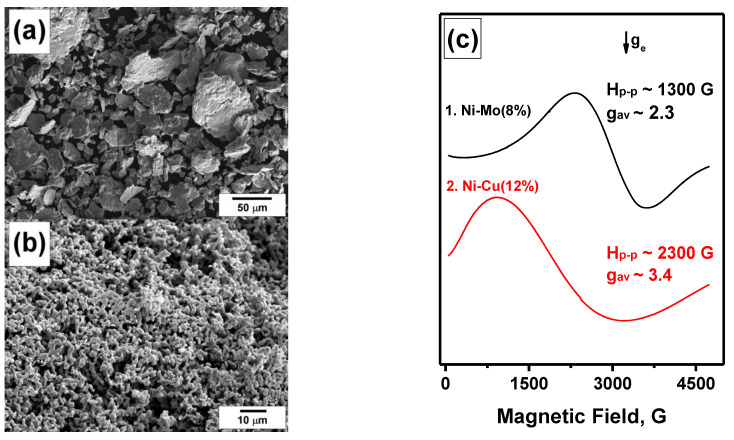
SEM images of the initial samples Ni-Cu (**a**) and Ni-Mo (**b**) and corresponding FMR spectra (**c**).

The other two samples studied in the present work, Ni-Cu(12%) and Ni-Mo(8%), are dispersed powders of significantly different morphologies, which is stipulated by the differences in their preparation routes. As seen in Figure 3a, the Ni-Cu(12%) sample is represented by micro-dispersed particles of a wide size distribution (10–150 μm). Since these particles underwent a drastic mechanical action during the MCA procedure, they have a rough surface with a number of cracks and splits. This morphology is typical of the materials prepared by the MCA method [41]. The SSA value for this sample was found to be 0.6 m^2^/g. The Ni-Mo(8%) sample was prepared by the reductive thermolysis technique [19,43,44]. Such a preparation method results in the formation of particles of a sponge-like morphology (Figure 3b) with a specific surface area of 0.2–0.5 m^2^/g.

The FMR spectra of these samples are represented by relatively wide singlet lines (Figure 3c). Note that a Curie temperature for the Ni-Mo alloy with the Mo content of 8% lies below zero (−150 °C). This means that the Ni-Mo(8%) alloy should not give any FMR signal in the initial state. However, a sufficiently intensive signal is seen in the spectrum. The observation of the FMR signal can be explained by the presence of nickel-reached domains within the composition of the particles, which can give such a signal. A detailed analysis of the spectrum evolution during the catalytic process is presented in Section 3.3.

Thus, both the previously reported data and the presented in this section results convincingly show that the FMR method can be successfully applied to study the evolution of nickel-containing catalysts taking place during the catalytic synthesis of CNF from various organic precursors. The high sensitivity of this method allows for observing the self-dispersion of the bulk metals and alloys in the course of the induction period when no carbon accumulation is registered [31]. Another interesting application of this technique is the possibility for a quantitative diagnostics of the non-uniformity of nickel alloys, for which the Curie temperature lies below the spectrum registration temperature.

### 3.2. Interaction of Ni-Cr Alloy with Halogenated Hydrocarbons under RAPET Conditions

As reported by Gedanken et al. [35,36,37,38,39,40], the RAPET conditions give a unique possibility to obtain new classes of structurally organized materials. A similar approach was applied in our recent works to study catalytic pyrolysis in the presence of bulk nickel alloys [32,33,34,45]. The proposed characterization method is based on the registration of the FMR spectra in an in situ mode. A sealed quartz ampule loaded with an alloy sample and organic substrate (Figure 1) was used as a reactor in these experiments. The ampule was stepwise heated to the desired temperature (up to 850 °C) and the FMR spectra were recorded after each temperature step.

The catalytic corrosion processes under RAPET conditions when nickel and its alloys interact with halogen-containing organic molecules are quite general and in many cases are accompanied by the formation of new structured nanomaterials–nickel halogenides [32,33,34]. Such compounds can be considered as intermediates in the processes of the submicron metal particle formation from bulk precursors during the synthesis of CNF from halogen-containing organic substrates in a flow-through reactor as well. In such processes, the fragmentation (disintegration) of the bulk metal is accompanied by the formation of sufficiently homogeneous submicron (200–300 nm) metal particles, on which the growth of CNF occurs. Catalytic corrosion processes involve oxidation-reduction steps during the interaction of nickel with a reaction medium. For halogen-containing compounds, this interaction can be described by the following scheme [33]:

Ni + 2RX → NiX_2_ + 2R (1)

NiX_2_ + H_2_ → Ni + 2HX (2)

where X is halogen contained in the initial organic substrate.

**Figure 4:**
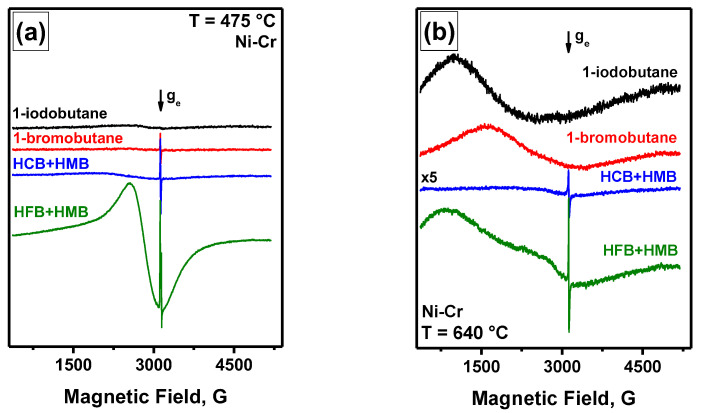
FMR spectra of nichrome interacting with various organic substrates under RAPET conditions at 475 °C (**a**) and 640 °C (**b**). The multiplier ×5 indicates the relative amplification coefficient used at registering the spectrum.

The results of the FMR comparative study on catalytic corrosion of nichrome interacting with various halogens (iodine, bromine, chlorine, and fluorine) under RAPET conditions are presented in Figure 4. The substrates were 1-iodobutane, 1-bromobutane, hexachlorobenzene (HCB), and hexafluorobenzene (HFB). In the last two cases, hexamethylbenzene (HMB) was loaded into the ampule as a hydrogen source for the reaction (2). As seen from the FMR spectra (Figure 4a), in the case of fluorine-containing medium, the formation of quite uniform ferromagnetic particles occurs already at 475 °C. The spectrum is characterized by the narrowest FMR signal (*H*_p-p_ = 550 G, *g* = 2.3), which indicates the formation of relatively small metal particles. An additional heating of the ampule at 640 °C results in changing the shape of the spectrum (Figure 4b). A significant enlargement of ferromagnetic particles can supposedly explain this. The appearance of an intensive narrow EPR signal in a *g*~*g*_e_ region typical for paramagnetic sites of coke should also be noted.

An unusual situation is observed for the chlorine-containing medium. A low-intensive FMR signal appears at the minimal temperature of 475 °C and then practically disappears at further heating. This can be connected with the formation of nickel chloride at elevated temperatures, which does not possess ferromagnetism.

These results clearly indicate the possibility to follow the evolution of the magnetic properties of nickel alloys by the FMR method during their interaction with various halogen-containing compounds. It should also be noted that the addition of HMB as a second component when using HFB and HCB as fluorine- and chlorine-containing compounds is due to the need to introduce hydrogen as a reducing agent. In the cases of individual substrates (HFB, HCB, and HMB) in similar experiments, no FMR signals have appeared throughout the studied temperature range.

It is obvious that other compounds that initiate catalytic corrosion of nichrome and contain, in particular, oxygen or nitrogen can act as an oxidizing agents in such reactions. As such an N-containing reagent, melamine can be used. Interestingly, the reaction of bulk nichrome with this reagent under RAPET conditions not only made it possible to detect the appearance of dispersed particles of a ferromagnetic metal but also produced CNF with a high nitrogen content [46].

Note that the FMR technique is also applicable to explore the state of the Ni-Cr alloy subjected to an acid etching procedure, which is accompanied by significant enrichment of the surface metal layer with nickel due to more efficient etching of chromium from the alloy [31].

### 3.3. Interaction of Ni-Cu(12%) and Ni-Mo(8%) Alloys with Polymers under RAPET Conditions

In Section 3.1, data on the initial state of the Ni-Cu and Ni-Mo catalysts used in this study were presented. It should be noted again that the homogeneous Ni-Mo alloy containing 8% of molybdenum should have a Curie temperature of −150 °C and should not possess ferromagnetism at room temperature. The appearance of a sufficiently intense FMR signal in the spectrum (Figure 3c) is most likely due to the heterogeneity of the alloy and the presence of nickel-rich particles in it. This section presents the evolution of Ni-Cu and Ni-Mo catalysts during their interaction with chlorine-containing (polyvinyl chloride) and chlorine-free (polyethylene) polymers. The samples were studied under RAPET conditions in a temperature range of 450–850 °C with a step of 100 °C. At each temperature point, the ampule was maintained for 2 h.

**Figure 5:**
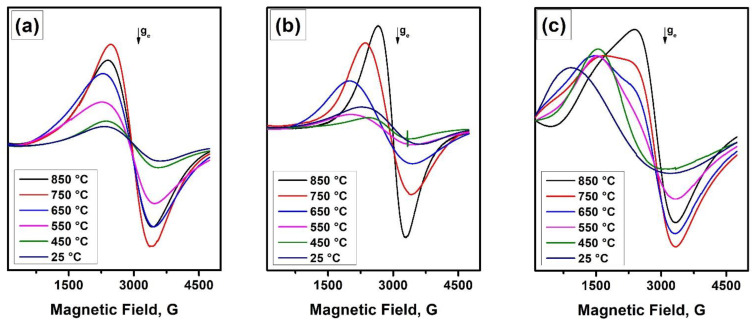
FMR spectra of Ni-Mo(8%) interacting with polyethylene (**a**) and polyvinyl chloride (**b**) under RAPET conditions at various temperatures. FMR spectrum of Ni-Cu(12%) interacting with polyvinyl chloride (**c**) under RAPET conditions at various temperatures.

The evolution of the FMR spectrum of the Ni-Mo(8%) sample during its interaction with polyethylene is shown in Figure 5a. As seen, the signal parameters *(g* and *H*_p-p_) are not practically changed with a temperature increase. Only a rise in the amplitude of the FMR signal is observed, which may indicate an increase in the depth of carbon erosion of the Ni-Mo alloy. At the same time, when polyvinyl chloride is used as a reagent (Figure 5b), there is a noticeable shift of the *g*-factor towards large fields and a significant decrease in the width of the signal (*H*_p-p_), similar to the evolution of the FMR spectra of nickel and its alloys described elsewhere [9,31].

Similar behavior was observed for the Ni-Cu(12%) alloy interacting with polyvinyl chloride. The Curie temperature for this alloy lies near +250 °C, therefore, a wide FMR signal is well seen at room temperature (Figure 3c). Figure 5c shows the evolution of the FMR spectra of this alloy during its interaction with polyvinyl chloride under RAPET conditions. With successive heating of the sample in a temperature range from 450 to 850 °C, the *g*-factor is shifted toward large fields. The results obtained for this sample also indicate a significant change in the magnetic properties of the initial alloy when interacting with a halogen-containing polymer.

**Figure 6:**
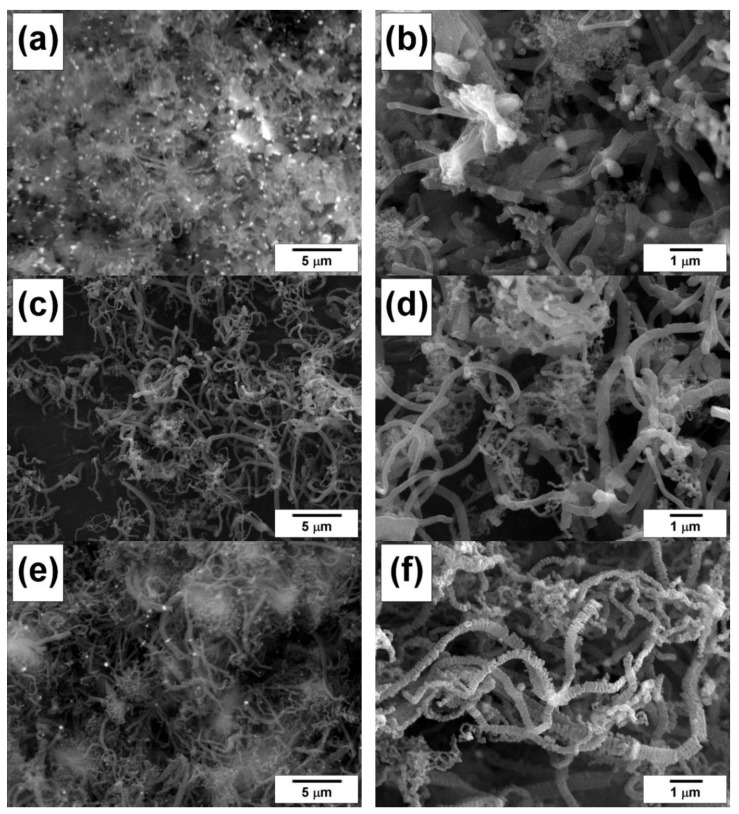
SEM images of the samples after the interaction of alloys with polymers under RAPET conditions: Ni-Mo(8%) with polyethylene at 550 °C (**a**) and 850 °C (**b**); Ni-Mo(8%) with polyvinyl chloride at 550 °C (**c**) and 850 °C (**d**); Ni-Cu(12%) with polyvinyl chloride at 550 °C (**e**) and 850 °C (**f**).

Figure 6 shows characteristic SEM images of Ni-Mo(8%) and Ni-Cu(12%) samples after their interaction with the investigated polymers in RAPET mode at temperatures of 550 and 850 °C. In almost all cases, it is possible to observe the simultaneous presence of the nanostructured carbon product along with the dispersed metal particles formed as a result of the disintegration of the initial alloy. The presence of dispersed particles can be evidently seen in Figure 6b–d. In the case of polyvinyl chloride decomposition, for both alloys, there is a tendency to form carbon deposits in the shape of thin flakes (Figure 6d–f).

Thus, the FMR and SEM data indicate an effective process of carbon erosion of Ni-Cu(12%) and Ni-Mo(8%) alloys during their interaction in a closed volume with polymer substrates (polyethylene, polyvinyl chloride). Deep disintegration of the alloys results in the appearance of dispersed alloy particles involved in the further deposition of nanostructured carbon during catalytic pyrolysis of the substrates.

### 3.4. Synthesis of CNFs over Ni-Cu(12%) and Ni-Mo(8%) Alloys in a Flow-Through Reactor

It should be emphasized that the Ni-Cu and Ni-Mo alloys investigated in the present work are important and effective catalysts for CNF synthesis. Exactly the processes of catalytic corrosion under the action of the reaction medium lead to the formation of catalytically active particles from the starting bulk alloys.

**Figure 7:**
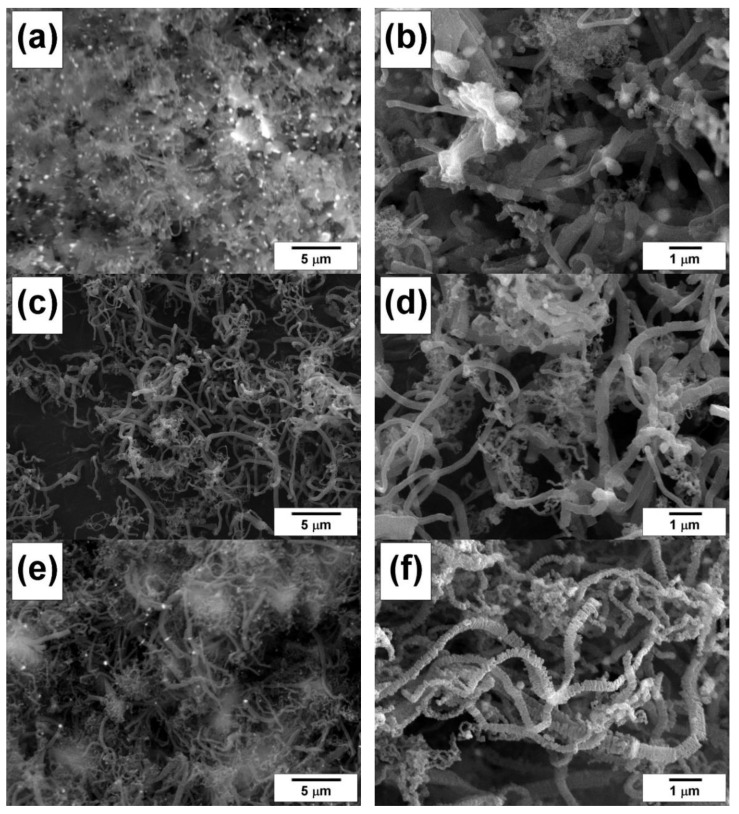
SEM images of the carbon product obtained via the interaction of alloys with the reaction mixture in a flow-through reactor: Ni-Cu(12%) with C_2_H_4_/H_2_/Ar at 550 °C for 1 min (**a**,**b**) and 30 min (**c**,**d**); Ni-Mo(8%) with C_2_H_4_Cl_2_/H_2_/Ar at 550 °C for 120 min (**e**,**f**).

Figure 7a,b show the result of the ethylene interaction with the Ni-Cu(12%) alloy at 550 °C for a short time (1 min). It can be seen that even after such a short time, bulk metal alloys (Figure 3a) undergo substantially complete disintegration to form dispersed particles (about 1 μm in size) catalyzing the further growth of CNF. Numerous active particles are evidently seen in SEM images in a backscattered-electron mode (Figure 7a,b). After 30 min of interaction with ethylene, long carbon filaments are formed, the diameter of which does not exceed 1 μm (Figure 7c,d).

Similar self-disintegration effects are observed in the case of the Ni-Mo(8%) alloy [20]. Figure 7e,f show the result of its interaction with DCE vapors at 550 °C. It is clearly seen that carbon filaments of predominantly submicron diameter (200–350 μm) having a pronounced segmented structure are formed under these conditions.

**Figure 8:**
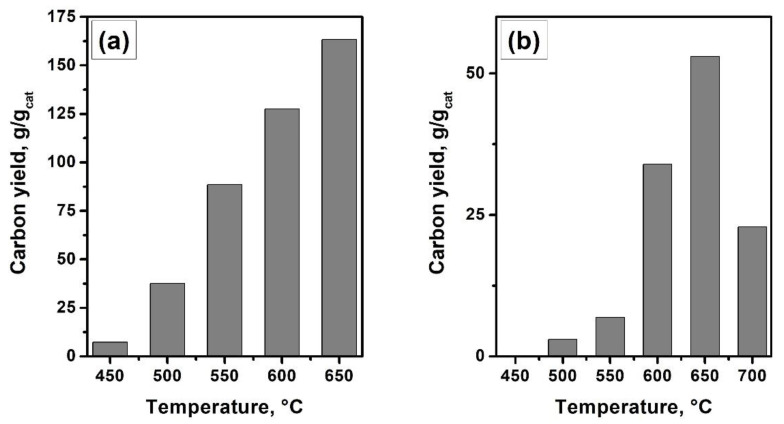
Temperature dependence of the carbon yield in a flow-through reactor: Ni-Cu(12%), C_2_H_4_/H_2_/Ar, 30 min (**a**); Ni-Mo(8%), C_2_H_4_Cl_2_/H_2_/Ar, 120 min (**b**).

**Figure 9:**
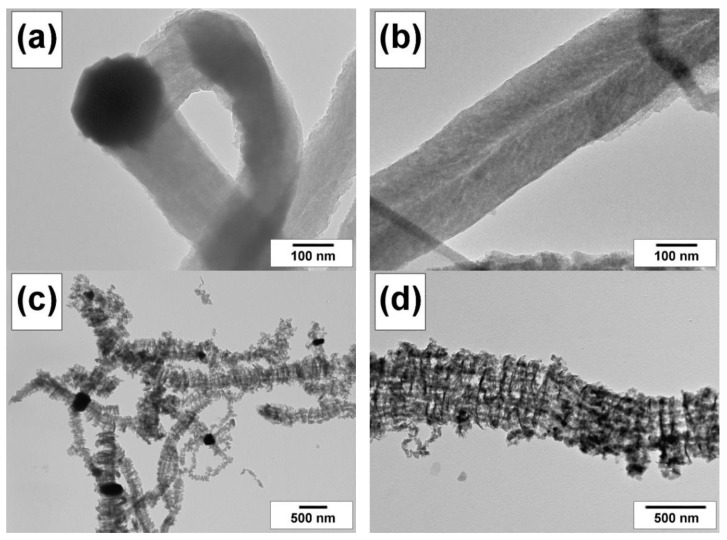
TEM images of the carbon product obtained via the interaction of alloys with the reaction mixture in a flow-through reactor: Ni-Cu(12%) with C_2_H_4_/H_2_/Ar at 600 °C for 30 min (**a**,**b**); Ni-Mo(8%) with C_2_H_4_Cl_2_/H_2_/Ar at 600 °C for 120 min (**c**,**d**).

Figure 8 demonstrates the dependence of the carbon yield on the temperature of the substrate decomposition over the self-organizing catalysts. In the cases of both reaction mixtures, C_2_H_4_/H_2_/Ar and C_2_H_4_Cl_2_/H_2_/Ar, there is an increase in the yield of CNF with a rise in the process temperature. In the case of ethylene decomposition at 650 °C, the productivity of the Ni-Cu(12%) catalyst after 30 min exceeds 160 g/g_cat_. When processing DCE over the Ni-Mo(8%) alloy, the carbon yield is several times lower (55 g/g_cat_ after 120 min of the experiment). This difference is caused by the presence of chlorine in the reaction mixture, which partially deactivates the catalyst by chemisorption on its surface. The presence of chlorine also affects the morphology of the resulting carbon material (Figure 7e,f and Figure 9c,d).

According to TEM data, the carbon nanofibers resulting from the ethylene decomposition have a tightly packed structure (Figure 9a,b). Both the types of packaging of graphene layers, the stacked (platelet) type (Figure 9a) and the coaxial-conic (fish-bone) type (Figure 9b), are equally found in the sample. In turn, the decomposition of the chlorine-substituted hydrocarbons leads to the formation of poorly ordered carbon filaments with a segmented structure (Figure 9c,d).

**Figure 10:**
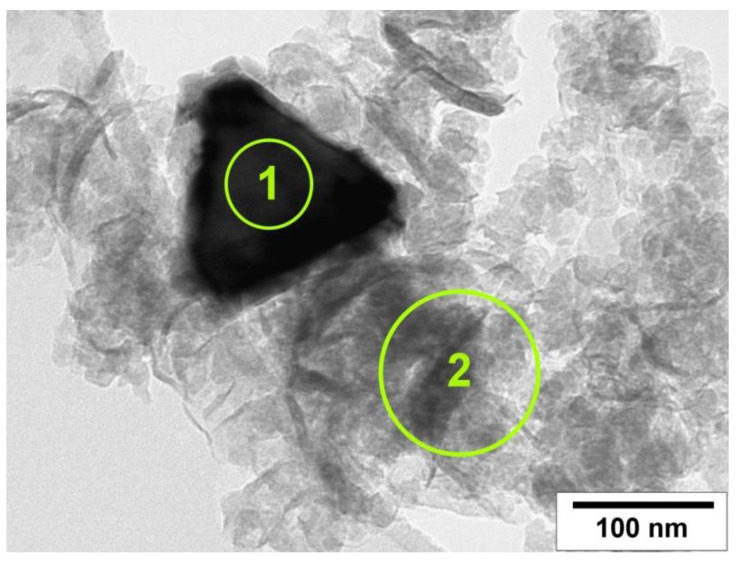
TEM image of the carbon product obtained via the interaction of Ni-Mo(8%) alloy with C_2_H_4_Cl_2_/H_2_/Ar at 600 °C in a flow-through reactor. The EDX analysis areas are highlighted by circles.

According to EDX data, the average concentration of Cl present on the surface of CNF is about 0.1 at.%, whereas the metallic particles exhibited a higher content of chlorine—up to 1 at.% (Figure 10, Table 2). The data obtained are in good agreement with the earlier works related to the synthesis of CNF from chlorinated hydrocarbons using different Ni-M alloys (M = Fe, Cu, Mo, etc.) [20,47,48]. It should be noted that the presence of halogen atoms over the surface of similarly prepared carbon nanomaterials was recently confirmed by means of X-ray photoelectron spectroscopy [19,49]. Thus, the chemisorbed chlorine species present on the surface of the active metallic particles exerts a determining impact on the formation of segmented carbon filaments due to a periodic blockage of their surface [18,20].

## 4. Conclusions

The present paper shows that the processes of self-organization of nickel-containing catalytic systems are accompanied by the formation of sufficiently homogeneous metal particles, acting as active sites in the CNF synthesis. It is important to mention that, in order to initiate the catalytic corrosion processes of nickel and its alloys, the presence of compounds capable of acting as both an oxidizing agent and a reducing agent in the reaction medium is a necessary condition. Thus, in the case of using halogen-containing molecules (HFB and HCB) as an oxidizer, hydrogen-containing molecules (in our case, HMB) must be added to the reaction medium to initiate such processes in RAPET mode.

It is very likely that the process of self-disintegration of nickel alloys studied within this work in a flow-through reactor during the pyrolysis of halogen-containing organic substances also proceeds through the formation of nickel halogenides as intermediates, which were directly detected under RAPET conditions among the reaction products.

The results presented in this work indicate a highly informative use of the FMR method for monitoring the evolution of nickel alloys in the reactions of catalytic pyrolysis of various organic molecules. It is believed that the proposed approaches can also be applied to investigate the processes of catalytic corrosion of Co- and Fe-containing catalysts widely used in the synthesis of structured carbon nanomaterials.

## Figures and Tables

**Table 1:** Reference data on a Curie temperature for nickel and its alloys [42].

#	Material	Curie temperature, °C
1	Ni	+360
2	Ni-Cr(23%)	−120
3	Ni-Cu(12%)	+250
4	Ni-Mo(8%)	−150

**Table 2:** EDX data for the Ni-Mo(8%) sample after its interaction with C_2_H_4_Cl_2_/H_2_/Ar at 600 °C in a flow-through reactor (see Figure 10).

Spectrum	Concentration, at.%			
	C	Cl	Ni	Mo
Area 1	-	0.8	94.5	4.7
Area 2	99.92	0.08	0	0

## Data Availability

Data are contained within the article.
